# White Dots as a Novel Marker of Diabetic Retinopathy Severity in Ultrawide Field Imaging

**DOI:** 10.1371/journal.pone.0165906

**Published:** 2016-11-03

**Authors:** Yoko Dodo, Tomoaki Murakami, Noriyuki Unoki, Ken Ogino, Akihito Uji, Shin Yoshitake, Nagahisa Yoshimura

**Affiliations:** From the Department of Ophthalmology and Visual Sciences, Kyoto University Graduate School of Medicine, Kyoto, Japan; University of Florida, UNITED STATES

## Abstract

**Purpose:**

To characterize white dots in diabetic retinopathy (DR) and their association with disease severity using ultra-wide-field scanning laser ophthalmoscopy.

**Methods:**

We randomly selected 125 eyes of 77 patients (25 eyes from individual categories of the international classification of DR severity) for which ultrawide field photographs were obtained. We characterized white dots, which were delineated by higher signal levels on green but not red laser images, and evaluated the relationship between the number of white dots and the international severity scale of DR.

**Results:**

Most white dots were located in nonperfused areas, and the number of total white dots was significantly correlated to that of dots in nonperfused areas. White dots corresponded to microaneurysms around the boundary between nonperfused areas and perfused areas or unknown lesions in nonperfused areas. Eyes with DR had significantly more white dots than those with no apparent retinopathy. The numbers of white dots in moderate nonproliferative diabetic retinopathy (NPDR) or more severe grades were significantly higher than in mild NPDR. The area under the receiver operating characteristics curve (AROC) analyses demonstrated that the number of white dots had the significance in the diagnosis of DR (0.908–0.986) and moderate NPDR or more severe grades (0.888–0.974).

**Conclusions:**

These data suggest the clinical relevance of white dots seen on ultrawide field images in the diagnosis of the severity of DR.

## Introduction

Diabetic retinopathy (DR), a leading cause of visual loss worldwide, may show angiogenic complications and may be complicated by diabetic macular edema (DME).[1–4] Among several DR classifications, the International Clinical Diabetic Retinopathy Disease Severity Scale that is based on evidence regarding progression to proliferative diabetic retinopathy (PDR) has been introduced.[5, 6] In addition, recent advances in surgical and medical interventions including anti-vascular endothelial growth factor (VEGF) therapy have improved visual prognosis in eyes with PDR or DME.[3, 7, 8] Improved diagnosis and treatment would provide greater benefits for patients with diabetes, although it is important to assess the clinical severity of DR correctly and pathogeneses in individual patients.

DR is a microvascular complication of diabetes, the first sign of which is the presence of microaneurysms.[5, 6] Diabetes disrupts the blood-retinal barrier, which is clinically represented by ophthalmoscopic findings such as retinal edema, intraretinal hemorrhages, and hard exudates or fluorescein leakage on fluorescein angiography (FA) images.[9, 10] As DR progresses, the dropout of retinal capillaries leads to ischemia or hypoxia in neuroglial tissues, and a concomitant increase in VEGF levels promotes angiogenic complications and exacerbates DME.[11, 12] It thus would be useful for clinicians if photographs alone could predict retinal capillary dropout in individual eyes with DR.[13, 14]

FA delineates retinal vascular lesions with higher contrast and sensitivity than color fundus photography and is the gold standard for assessing nonperfused areas. However, because FA is invasive, alternative methods should be developed.[15, 16] The international classification of DR severity recommends that clinicians evaluate fundus findings using the 4-2-1 rule, i.e., multiple retinal hemorrhages, venous beading, and intraretinal microvascular abnormalities (IRMAs) as biomarkers for predicting progression to PDR.[5] Intraretinal hemorrhages may represent damage in the capillary beds to some extent. Nonperfused areas often are seen around venous beading. IRMAs, which are either shunt vessels or intraretinal neovascularization, are accompanied by nonperfused areas in the peripheral retina. If present, additional markers of the nonperfused areas would help clinicians better understand the pathogenesis in each patient.

In the current study, we documented for the first time the characteristics of white dots in DR using ultra-wide-field scanning laser ophthalmoscopy (SLO) and showed their diagnostic significance in DR severity and the association with nonperfused areas.

## Materials and Methods

### Patients

All research and measurements adhered to the tenets of the Declaration of Helsinki. The Institutional Review Board and Ethics Committee of Kyoto University Graduate School of Medicine approved the study protocol and all participants provided written informed consent. We retrospectively reviewed 125 eyes of 125 patients (age range, 26–82 years; mean, 63.2±12.0 years) for whom ultra-wide-field photographs of sufficient quality were obtained at the Department of Ophthalmology of Kyoto University Hospital from November 2011 to December 2013. We randomly selected 25 eyes from each category of the international severity scale of DR, i.e., no apparent retinopathy; mild, moderate, and severe nonproliferative diabetic retinopathy (NPDR); and PDR. We also obtained FA and spectral-domain optical coherence tomography (SD-OCT) images of 74 eyes (24 eyes with moderate NPDR, 25 with severe NPDR, and 25 with PDR) and compared them to the findings on the ultrawide field color photographs. The main exclusion criteria were the presence of vitreous hemorrhage (and/or preretinal hemorrhage), tractional retinal detachment, or any other chorioretinal diseases, and a history of any treatment for DR or DME including retinal photocoagulation, cataract surgery within 3 months, or any major surgery other than cataract extraction. Images which were affected by media opacities and the cilia were excluded from this study. Diabetic patients often suffer from systemic hypertension which might influence retinal vasculature. We thus evaluated 25 unaffected fellow eyes of 25 age-matched retinal vein occlusion patients as nondiabetic control subjects (age range, 29–92 years; mean, 63.5±14.2 years) after the exclusion according to the criteria above (Table 1).

**Table 1:** Systemic characteristics of control subjects and diabetic patients.

		Healthy subjects (n = 25)	Diabetic patients (n = 125)	*P*-value
Age (years)		63.5±14.2	63.2±12.0	0.960
Gender	male	13	80	0.269
	female	12	45	
HbA1c (%)		-	8.05±2.12	-
Hypertension	present	15	78	0.825
	absent	10	47	

*P*-value; comparison between healthy subjects and diabetic patients. Student t-test for the comparison of age; Fisher’s exact test for the comparison of gender and hypertension.

### Ultrawide field imaging

Two consecutive fundus images were obtained and applied to two steps of the image processing to discriminate white dots from hard exudates, drusen, and dot-like artifacts, followed by the manual counts of such lesions. We obtained two consecutive ultrawide field images (200-degree retinal fields Optomap plus [UWF200]) using ultrawide field SLO (Optos200Tx, Optos PLC, Dunfermline, Scotland) after mydriasis was achieved with topical instillation of 2.5% phenylephrine hydrochloride and 1.0% tropicamide.[17] After exported as TIFF files, the fundus images with actual pixel resolution opened in the image processing software (Adobe Photoshop, Adobe systems Inc.) were evaluated on a diagnostic monitor (FlexScan SX2762W, EIZO Co., Ishikawa, Japan) under the default parameter settings.

**Fig 1:**
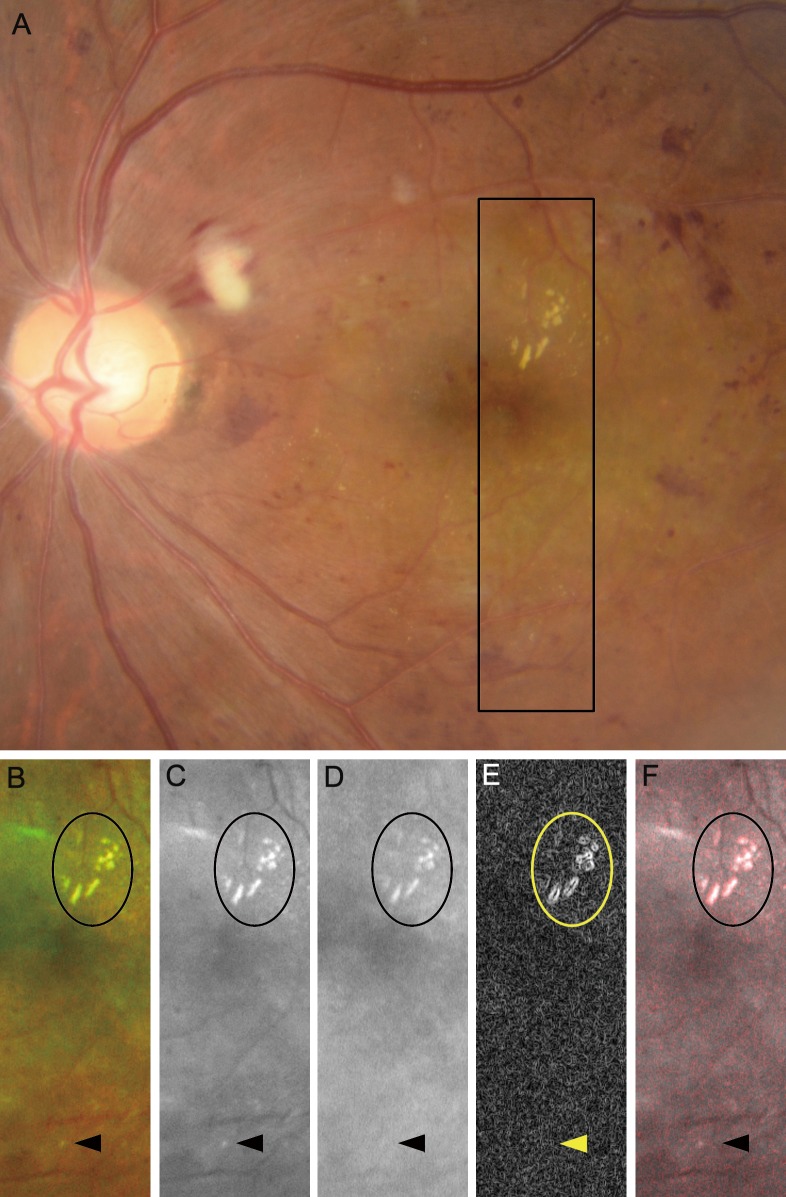
Different appearances of white dots and typical hard exudates in diabetic retinopathy. (A) color fundus photograph shows yellow and metallic hard exudates and white dots (rectangular area). (B, C) Pseudocolor and green-channel ultrawide field scanning laser ophthalmoscopy images. (D) The red-channel image shows that hard exudates and not white dots are accompanied by higher signal intensity. (E) The margin of the hard exudates is delineated clearly with the Find Edges function of the ImageJ software compared to that of the white dots. (F) A merged image comprised of the panels C (gray) and E (red). Oval areas = hard exudates; arrowheads = white dots.

We defined fine and well-demarcated whitish dots as white dots after discriminating them from yellowish deposits, hard exudates and drusen. First, we compared the ultrawide field SLO images to 45-degree color fundus photograph (3,216 x 2,136 pixels) centering on the fovea using a fundus camera (TRC-50LX, Topcon, Tokyo, Japan), because we had to clarify that white dots on the SLO images corresponded to those on fundus examinations. We then differentiated white dots from typical hard exudates, drusen, and dot-like artifacts. White dots had higher signal levels in the green-channel images (532 nm), whereas the signals were absent or faint in the red-channel images (633 nm). In contrast, typical yellowish and metallic hard exudates or drusen had high signal intensities in both channels (Fig 1). Considering the different signal levels in the red-channel images, we differentiated the white dots from hard exudates. Briefly, the margin of the dots in the red-channel images was delineated using the Find Edges function of the ImageJ software (National Institutes of Health, Bethesda, Maryland) and merged onto the dots in the green-channel images using the Colour Merge function of the ImageJ plugin. In the merged images, the edges in the red-channel images appeared as red and the green-channel images as grayscale. Hard exudates appeared to be white or gray dots enclosed by red borders. We defined the dots without edges as white dots or dot-like artifacts (Fig 1).

**Fig 2:**
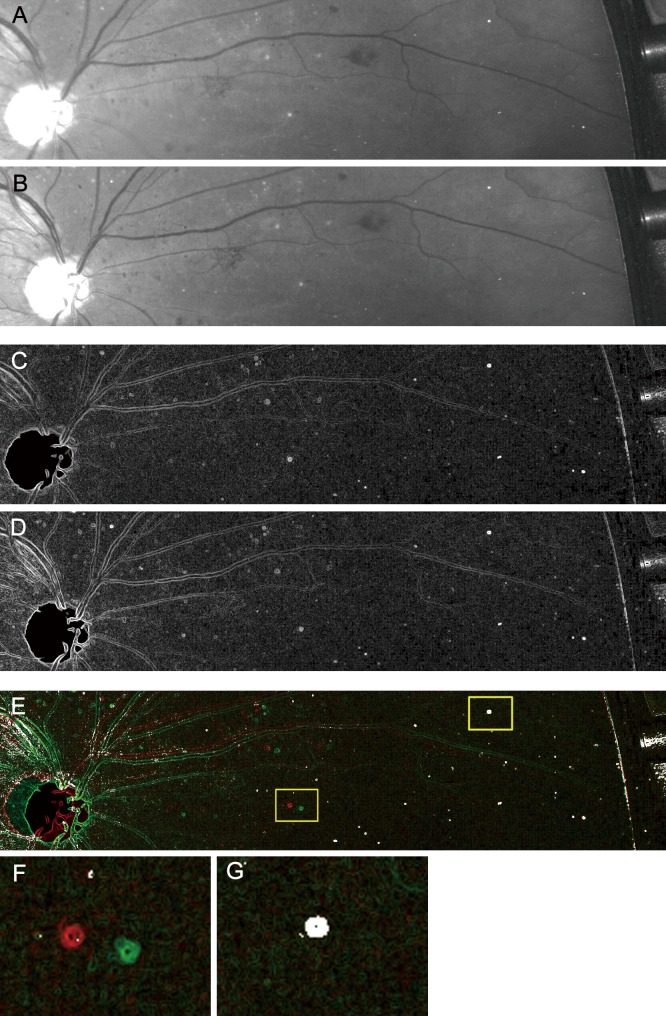
Differentiation of the white dots from the dot-like artifacts on ultrawide field imaging. (A, B) Raw images of two sequential green-channel images. (C, D) The Find Edges function of the ImageJ software is applied to the images. (E) The co-localization highlighter function of the ImageJ plugin is then applied. (F, G) The magnified images of the left and right squares in panel E, respectively. (F) The twin dots (green and red) correspond to the white dots. (G) Dot-like artifacts are co-localized completely.

As the second step, we also compared two consecutive green-channel images to discriminate the white dots from dot-like artifacts from this new instrument itself. Two consecutive images were unintentionally captured from slightly different viewpoints, meaning that the artifacts were at the same position in the different images, whereas the white dots had different locations (Fig 2). We applied the Find Edges function followed by the Colocalization Highlighter function of the ImageJ plugin and determined that the co-localized dots corresponded to the dot-like artifacts, whereas twin dots with a higher signal intensity were white dots per se. After we excluded hard exudates, drusen, and dot-like artifacts using two steps of the image processing as describe above, we manually counted the white dots in the entire areas acquired by ultra-wide-field SLO. We confirmed the agreement of the numbers of dots obtained by two independent masked graders (intraclass correlation coefficient, 0.986), and the average was applied for further analysis.

**Fig 3:**
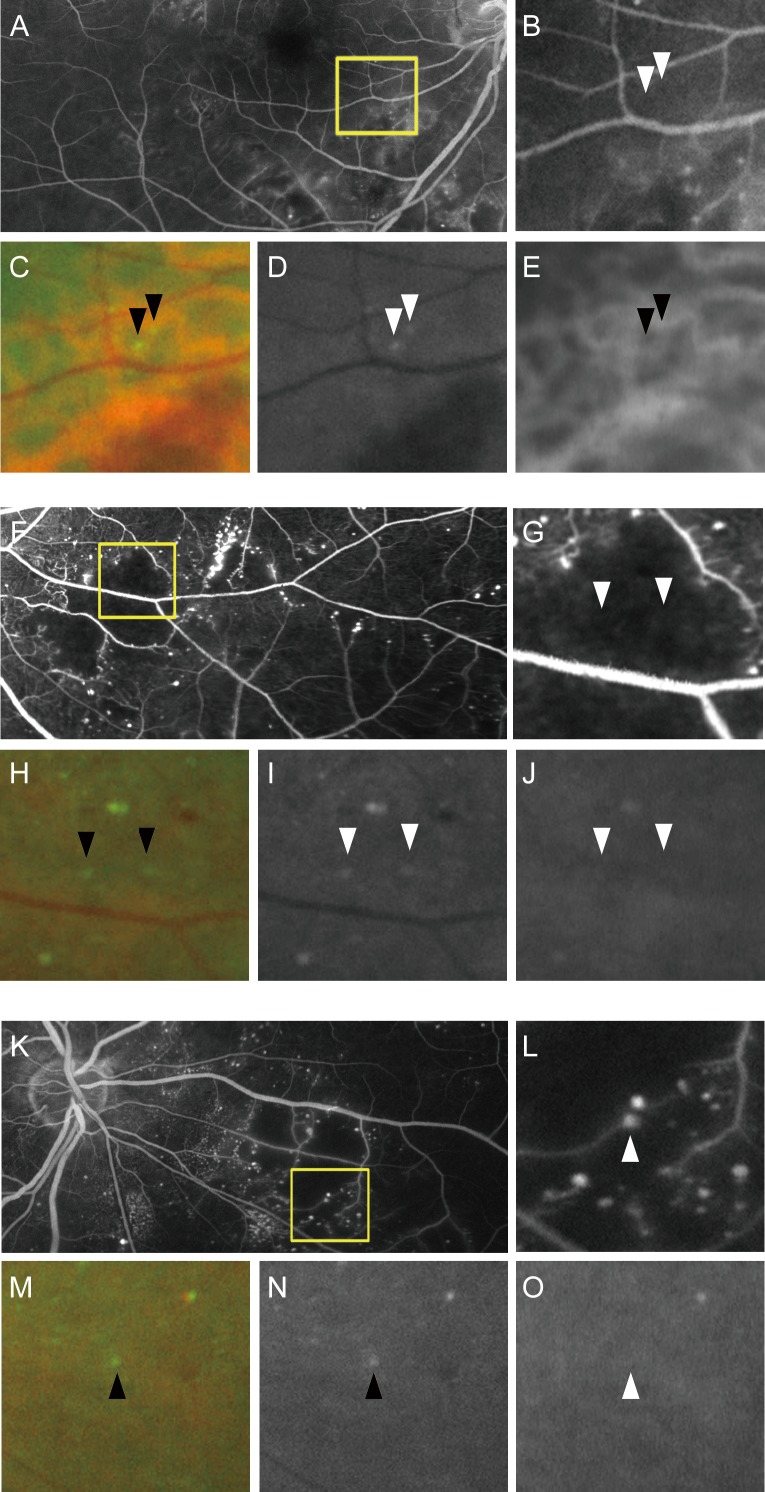
White dots are seen in the perfused or nonperfused areas in diabetic retinopathy. White dots are delineated rarely in the typical perfused areas (A-E) and mainly in the typical nonperfused areas (F-J). Some white dots correspond to microaneurysms in the areas at the border between the typical perfused areas and nonperfused areas (K-O). (A, F, K) Fluorescein angiography and (B, G, L) its magnified image. (C, H, M) Pseudocolor, (D, I, N) green-channel, and (E, J, O) red-channel ultrawide field scanning laser ophthalmoscopy images corresponding to the magnified FA images (B, G, L), respectively. Arrowheads = white dots.

Early- and late-phase FA images were acquired using the Optos200Tx. We compared the white dots to two FA findings, microaneurysms and nonperfused areas. We checked the co-localization of the white dots and microaneurysms, which were delineated as hyperfluorescent dot-like lesions in either the early- or late-phase images or both (Fig 3). Most white dots were in the nonperfused areas and did not have corresponding findings on the FA images (Fig 3).

We then investigated the relationship between the white dots and nonperfused areas.

The areas with neither capillaries nor background fluorescence were first defined as the typical nonperfused areas in the current study (Fig 3G). The lower resolution of this ultrawide field imaging system prompted us to define the areas without distinct capillaries but with higher background fluorescence as the typical perfused areas (Fig 3B). We often observed the areas with lower background fluorescence and dilated and sparse capillaries after the vascular remodeling. We thus included the areas with no or lower background fluorescence and the boundary between the typical nonperfused areas and the typical perfused areas (Fig 3L) into the nonperfused areas in the current study. We further counted white dots in the nonperfused areas.

We further counted red spots which contain microaneurysms and dot-like intraretinal hemorrhages on the ultrawide field SLO images. The numbers of red spots identified by two masked independent graders showed higher agreement (ICC = 0.956). Further, the comparative study using the FA images allowed us to discriminate microaneurysms from dot-like intraretinal hemorrhages.

### Optical coherence tomography

We obtained retinal sectional images using SD-OCT (Spectralis OCT, Heidelberg Engineering, Heidelberg, Germany). The raster scan mode (17 lines x 20 degree) as installed by manufacturers was applied centering on the fovea, and white dots were by chance dissected. Their reflectivity and morphologies on the sectional images were characterized.

### Statistical analysis

Two independent masked retinal specialists quantified the number of retinal hemorrhages and/or microaneurysms, and the mean number was used for further analyses. Two independent retinal specialists also assessed the qualitative findings on the ultra-wide-field images in a masked fashion, and the third specialists settled any disagreements.[17]

The results are expressed as the mean ± standard deviation for the parameters with normal distributions and equal variance or otherwise the median (interquartile range [IQR]). Student t-test was applied for the datasets with normal distributions and equal variance, and the differences in the sampling distribution was evaluated using Fisher’s exact test. The data were analyzed using Kruskal-Wallis test with Bonferroni correction for populations with non-normal distributions or unequal variance. Spearman rank correlation coefficients were employed to determine statistical significance. The areas under the receiver operating characteristic curve (AROC) were calculated to validate the DR discriminating power of the total number of white dots and red spots. Briefly, we calculated the sensitivity and specificity for individual DR severity grades or more according to the number of white dots, and the receiver operating characteristic (ROC) curve was generated. The repeatability between two independent retinal specialists was evaluated by ICC. These statistical analyses were performed using commercial software (PASW Statistics, version 18; SPSS Inc., Chicago, IL). P<0.05 was considered significant.

## Results

### Characteristics of the white dots

The patients characteristics in the current study were shown in Table 1. We first counted white dots on ultrawide field SLO images in eyes of subjects with and without diabetes mellitus. Seventeen of 25 nondiabetic control eyes had no white dots, and only one dot was delineated in 7 eyes. Eyes of diabetic patients with or without DR had more white dots than eyes of healthy subjects (14.5 [IQR, 7–25.25] or 1 [IQR, 0–2] vs. 0 [IQR, 0–1]; *P*<0.001 or *P* = 0.040, respectively), suggesting that white dots are one of clinical findings of DR.

In order to validate the clinical feasibility of the SLO images and the following image processing, we compared white dots on color fundus photograph to those on ultrawide field SLO images in 48 eyes on which both images were obtained on the same day. One hundred twenty-two white dots were delineated on either 45-degree fundus photograph centering on the fovea or the SLO imaging system, and 120 dots corresponded to each other (ICC = 0.984). It prompted us to evaluate the location of white dots on ultrawide field SLO images. After excluding dot-like artifacts and hard exudates, we observed means of 6 (IQR, 2–11), 2 (IQR, 0–5), 2 (IQR, 0–6), and 1 (IQR, 0–3) white dots in the superonasal, inferonasal, superotemporal, and inferotemporal quadrants, respectively, in 100 eyes with DR. The mean numbers of white dots in the central fields within 30 degrees, the Early Treatment Diabetic Retinopathy Study (ETDRS) seven standard 30-degree fields, and the peripheral fields (outside the ETDRS seven standard 30-degree fields) were 0 (IQR, 0–1), 8 (IQR, 3–18), and 3 (IQR, 1–6.25) respectively, suggesting the potential use of ultrawide field imaging to evaluate white dots.[17, 18]

**Fig 4:**
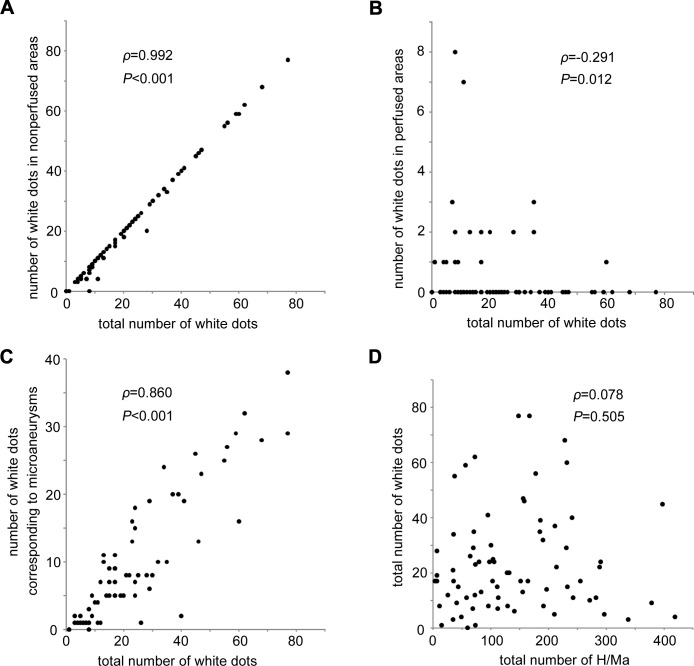
Quantitative analyses of white dots in 74 eyes on whom fluorescein angiography images were acquired. Association of the total number of white dots with the number of white dots in the nonperfused areas (A) or perfused areas (B), the number of white dots corresponding to microaneurysms on fluorescein angiography images (C), and the number of retinal hemorrhages and/or microaneurysms (H/Ma; D).

**Fig 5:**
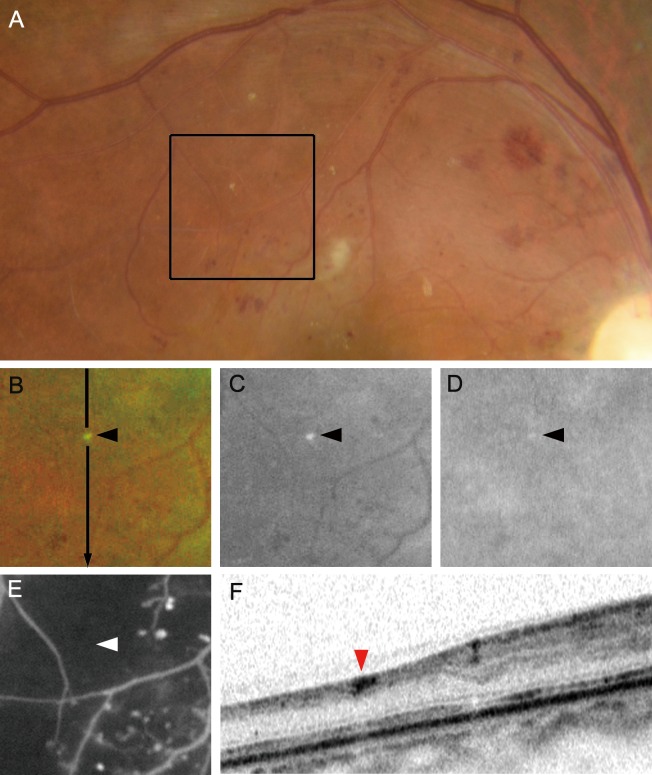
A white dot in the typical nonperfused area in diabetic retinopathy. A white dot seen in a color fundus photograph (A), pseudocolor image (B), green-channel (C), and red-channel (D) on ultrawide field scanning laser ophthalmoscopy images is seen in the typical nonperfused areas on fluorescein angiography image (E). (B-E) The magnified images corresponding to the black rectangle in panel A. (F) Optical coherence tomography image dissecting along the arrow in panel B shows a lesion with high reflectivity in the inner retinal layers. Arrowheads = white dots.

We then characterized the white dots in 74 eyes with DR compared to the lesions on the FA images. Most of white dots were delineated in nonperfused areas, and they were significantly correlated to total number of white dots (*ρ* = 0.992, *P*<0.001; Fig 4), whereas white dots in the perfused areas were modestly and negatively related to total number of dots (*ρ* = -0.291, *P* = 0.012; Fig 4). OCT images by chance delineated that several white dots corresponded to polygonal lesions with higher OCT reflectivity in the inner retinal layers in the typical nonperfused areas (Fig 5). Comparative studies further elucidated that that 31.6% (IQR, 21.2%-52.0%) of the white dots corresponded to microaneurysms, which appeared as hyperfluorescent dots on the FA images, and most white dots around the boundary of nonperfused areas corresponded to microaneurysms (Fig 3).[19–21] The total number of white dots was correlated significantly with the number of white dots corresponding to microaneurysms on FA images (*ρ* = 0.860, *P*<0.001) but not with the number of H/Ma on the SLO images (*ρ* = 0.078, *P* = 0.505; Fig 4). We sometimes observed white dots with no definite findings on the FA or OCT images; these dots should be characterized in future studies.

### Association between white dots and severity of DR

**Fig 6:**
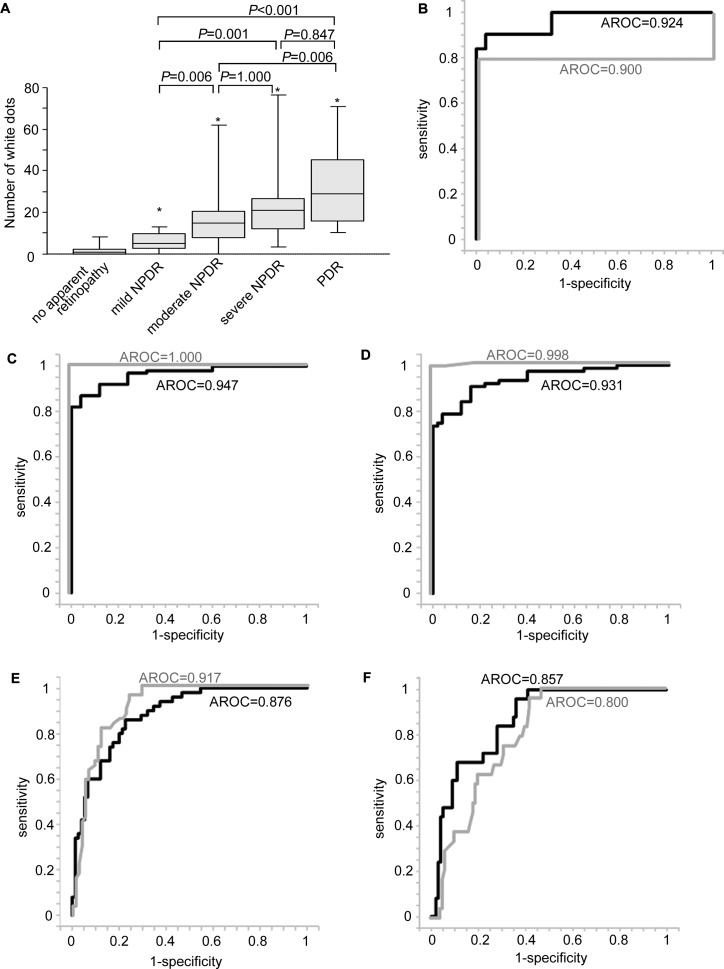
Clinical significance of white dots in the diagnosis of diabetic retinopathy severity. (A) The number of white dots (box plot) in individual grades of the international severity scale of diabetic retinopathy. *, *P*<0.001 vs. no apparent retinopathy. (B) The receiver operating characteristic (ROC) curve for diabetic retinopathy (DR) diagnosis of the total number of white dots or red spots discriminating 125 eyes of diabetic patients from 25 nondiabetic control subjects. The ROC curve for DR severity diagnoses (mild nonproliferative diabetic retinopathy [NPDR] or more severe [C], moderate NPDR or more severe [D], severe NPDR or more severe [E], and proliferative diabetic retinopathy [PDR] [F]) of the total number of white dots or red spots in 125 eyes of diabetic patients. AROC = the area under the receiver operating characteristic curve. black line = ROC curve of white dots; gray line = ROC curve of red spots.

We investigated the relationship between the white dots and the international classification of DR severity.[5] The mean number of white dots in 100 eyes with DR was significantly greater than that in 25 eyes with no apparent retinopathy (*P*<0.001). The numbers of white dots increased gradually with the severity of DR; eyes with mild NPDR had fewer white dots than those with moderate NPDR, severe NPDR, and PDR (*P* = 0.006, *P* = 0.001, and *P*<0.001, respectively; Fig 6A).

We then estimated the diagnostic significance of white dots in DR. Considering the sensitivity and specificity, the number of white dots had diagnostic significance to discriminate diabetic patients from the control subjects (AROC = 0.883–0.965; Fig 6B). In addition, AROC for mild NPDR or moderate NPDR had distinct ranges (0.908–0.986 or 0.888–0.974, respectively), whereas AROC for white dots showed modest accuracy for the prediction of severe NPDR or PDR (0.817–0.935 or 0.787–0.927, respectively; Fig 6). AROC for DR severity diagnosis of the total number of red spots was better than that of white dots’ number to discriminate mild NPDR, moderate NPDR, and severe NPDR (AROC = 1.000, *P*<0.001; AROC = 0.995–1.000, *P*<0.001; 0.869–0.965, *P* = 0.003, respectively), compared to no differences in the AROC for discriminating PDR (AROC = 0.720–0.879, *P* = 0.163).

We further investigated the association of white dots with systemic factors, and found that there was the negative and modest correlation between age and the number of white dots (*ρ* = -0.261, *P* = 0.004), whereas other parameters including gender, hemoglobin A1c, and systemic hypertension did not show the association (P = 0.990; *ρ* = -0.030, *P* = 0.760; *P* = 0.941).

## Discussion

The current study documented white dots as a marker of DR severity for the first time using ultrawide field SLO with green and red channels. White dots were observed in eyes of diabetic patients with and without DR, and the number of white dots gradually increased according to the DR severity. The AROC analyses demonstrated that the number of white dots had the significance in the diagnosis of DR and moderate NPDR or more severe grades. Intriguingly, white dots were observed in eyes with no apparent retinopathy, and the AROC analyses suggest that white dots on fundus images have clinical utility for the diagnosis of diabetes mellitus to some extent. However, white dots do not seem to be very specific for DR, since they are also found in other retinal vascular diseases including old retinal vein occlusion (data not shown).

Microaneurysms, multiple intraretinal hemorrhages, venous beading, IRMAs, and neovascularization are the markers of gold standard for this retinal vascular disease, although it often is difficult to detect these reddish findings on the red-orange fundus. In contrast, white dots appeared to be distinct on the ultrawide field SLO images, and would provide supportive information in the diagnosis of DR severity. Considering the FA and OCT findings, we speculated that despite their homogeneous morphology, the white dots corresponded to a few kinds of lesions, i.e., whitish microaneurysms around the boundary of nonperfused areas and the unknown lesions in the typical nonperfused areas. Anyway, white dots suggest the presence of nonperfused areas, which would support the association between the number of white dots and DR severity. There was no association between retinal hemorrhage and white dots, which is compatible to that there are few retinal hemorrhages in the typical nonperfused areas indicated by white dots. Patients with PDR sometimes had many white dots but not many retinal hemorrhages in this study. We thus speculate that white dots and retinal hemorrhages are complementary predictors of DR severity.

The international severity scale proposed the diagnostic significance of vascular lesions, i.e., multiple retinal hemorrhages, venous beading, and IRMA. However, aggregated retinal hemorrhages make it difficult to count themselves, and venous beading or IRMA develops less frequently. In contrast, it may be more feasible to clinically evaluate and count white dots in eyes with DR, because the findings with higher contrast on the dual SLO images are definite and simple, compared to red spots containing microaneurysms and retinal hemorrhages on the reddish fundus background. The green and red channels in the Optos200Tx allowed discrimination between the white dots and typical hard exudates. In addition, since ultrawide field imaging can visualize most retinal areas, this system has potential for evaluating fundus findings in DR.[17, 22, 23]

We further investigated the characteristics of the white dots compared to vascular lesions on FA images and found that some white dots were microaneurysms. Previous reports have described the various appearances of the microaneurysms, including whitish or reddish ones, which depended on the presence of red or white blood cells, the properties of the vascular walls, or the stages in fibrotic maturation.[19–21, 24] Further evaluation using adaptive optics imaging may elucidate the relationship between the microaneurysms of different colors and the flow of blood components.[25] In addition, most whitish microaneurysms were in the areas with low capillary density or around the boundary between the typical nonperfused areas and the typical perfused areas, which prompted us to speculate that these white dots may be developed during capillary dropout, because the disrupted flow of blood components, including erythrocytes, may affect the color of the microaneurysms. It had been reported that the numbers of microaneurysms were associated with progression of DR, which may lead us to plan a longitudinal study.[26, 27]

We could not determine the pathohistological lesions corresponding to white dots in the current study. Most white dots around the margin of the nonperfused areas were whitish microaneurysms, whereas there were no publications describing pathohistological lesions corresponding to the white dots in the nonperfused areas. Some white dots corresponded to lesions with higher OCT reflectivity in the inner retinal layers (Fig 5). The size, shape, and location of these lesions differed from the hyperreflective foci documented by Bolz and associates.[28] Pathohistologic studies have reported hyaline deposition and thinning of the inner retinal layers in nonperfused areas of DR, which might explain some lesions.[29] Considering the components of the retinal tissues, several other possibilities are that the lesions are remnants of thicker vascular walls, apoptotic aggregation of ganglion cells in the inner retinal layer, or changes in Müller cells.[29–31] Further clinicopathologic analyses would elucidate the cellular or histologic changes corresponding to white dots.

Although we excluded typical hard exudates, white dots in color photographs rarely corresponded to optical coherence tomographic hyperreflective foci mainly around the outer plexiform layer in perfused areas. It was reported that hard exudates appeared to be accumulated hyperreflective foci on OCT images.[28] The authors speculated that hyperreflective foci may be precursors of hard exudates or lipid-laden macrophages. Another recent publication reported that hyperreflective spots in eyes with or without DR may correspond to glial changes caused by diabetes.[32] Taken together, we hypothesized that these dots were on the same spectrum of yellow and metallic hard exudates.

The current study was limited in the evaluation and enumeration of the white dots seen on the ultrawide field SLO images. We often saw dot-like artifacts in the fundus images obtained by this instrument compared to classic photographic images. Distortion of the retinal lesions in the periphery also prevented us from correctly evaluating their shapes and sizes. The second limitation was that there were no significant differences in the numbers of white dots between eyes with moderate NPDR and severe NPDR, suggesting difficulty in determining the thresholds among these categories. An additional concern is the control subject. Diabetic patients often suffer from several systemic diseases as well, and we had difficulty in finding ideal control subjects. Since the current study was cross-sectional and retrospective, we could not conclude whether the white dots predict progression to PDR, and further longitudinal study should be planned to determine the diagnostic significance.

## Conclusions

In the current study, we documented the characteristics and the clinical relevance of white dots in DR on ultrawide field SLO images, which would support improved diagnosis of the severity of DR.
